# 25th Annual Meeting of the German Society of Newborn Screening

**DOI:** 10.3390/ijns4020017

**Published:** 2018-06-04

**Authors:** Vassiliki Konstantopoulou, Maximilian Zeyda

**Affiliations:** Austrian Newborn Screening and Metabolic Lab, Department of Pediatrics and Adolescent Medicine, Medical University of Vienna, Währinger Gürtel 18-20, A-1090 Vienna, Austria

**Keywords:** newborn screening, psychology, recalls, techniques, primary immunodeficiencies, severe combined immunodeficiencies (SCID), vitamin disorders

## Abstract

From 15–16 June 2018, the 25th Annual Meeting of the German Society for Newborn Screening (Deutsche Gesellschaft für Neugeborenenscreening, DGNS) was held at the Van Swieten Hall of the Medical University of Vienna, Vienna, Austria. For the first time, this annual meeting was held outside Germany and organized by Maximilian Zeyda, PhD and Vassiliki Konstantopoulou, MD (conference presidents), directors of the Austrian Newborn Screening located at the Dept. of Pediatrics at the Medical University of Vienna. A local scientific board formed by Maximilian Zeyda and Vassiliki Konstantopoulou selected presentations from abstracts that were submitted by scientists of 7 countries, highlighting one purpose of this meeting, which was to foster contact and exchange of newborn screening labs of central European countries. Abstracts of invited lectures, oral communications, and posters presented during the meeting are collected in this report.

## 1. Aim and Scope of the Meeting

The theme of the meeting “review-insights-outlook” allowed the program to highlight achievements such as expanded newborn and toxoplasmosis screenings in Austria, to focus on present challenges such as false-positives, psychological aspects, and technical progress, and to discuss possible future developments. According to the submitted abstracts, vitamin disorders emerged as currently much discussed, including vitamin B12 deficiency. The meeting also included the general assembly of the DGNS, and the National Newborn Screening Report 2016 for Germany was presented by the president of the DGNS, Dr. med. Uta Nennstiel. The main topic of the second day of the meeting was enabling newborn screening for conditions not yet screened, with an emphasis on primary immunodeficiencies. Therefore, international experts in SCID screening shared their experiences with the attendees.

## 2. Lectures

### L-01. First Results from a Study for the Evaluation of 26 Additional Target Disorders for the German Newborn Screening Panel

GramerGwendolynFang-HoffmannJunminFeyhPatrikKlinkeGlynisMonostoriPeterOkunJürgen G.HoffmannGeorg F.University Hospital Heidelberg, Centre for Paediatric and Adolescent Medicine, Division for Neuropaediatrics and Metabolic Medicine, Im Neuenheimer Feld 430, 69120 Heidelberg, Germany

Until March 2018 newborn screening in Germany included 15 target disorders. Recently tyrosinaemia type I has been added as 16th disorder to the German newborn screening panel. Diagnostic improvements suggest a further extension of the panel. Since August 2016 a prospective study evaluating 26 additional target disorders (25 metabolic disorders and vitamin B_12_-deficiency) in addition to the German screening panel is performed at the Newborn Screening Centre Heidelberg, using second-tier strategies for 15 of the additional target disorders. From August 2016 until April 2018 126.471 children participated in the study. The participation rate was 64.4% of our screening cohort in 2018. Second-tier analyses were performed in 6.9% of samples. The recall rate was very low with 0.1% for the additional target disorders. Target disorders from the study panel were confirmed in 28 children: 1 HMG-CoA-lyase-deficiency, 1 citrullinaemia type I, 3 multiple acyl-CoA dehydrogenase-deficiency, 1 MTHFR-deficiency, 2 OTC-deficiency, and 21 children with maternal vitamin B_12_-deficiency (one child with MAD-deficiency + vitamin B_12_ deficiency). All mothers of children with vitamin B_12_-deficiency were offered standardized work-up for vitamin B_12_-deficiency and were referred to internal medicine for further diagnostics and treatment if indicated. None of the mothers who were evaluated adhered to a vegan or vegetarian diet. Vitamin B_12_-deficiency in the mothers was caused by nutritional problems during pregnancy or suspected undetected dysfunctions of gastrointestinal absorption. No case of tyrosinaemia type I has been confirmed since August 2016 using succinylacetone in dried blood spots as primary marker. Two children with slightly elevated succinylacetone in newborn screening and traces of succinylacetone in urine are currently being evaluated for the differential diagnosis maleylacetoacetate isomerase deficiency. Concerning short-term outcome of the identified patients 25 were asymptomatic at time of diagnosis, 3 were symptomatic (2 patients with OTC-deficiency and 1 with severe MAD-deficiency). Two of the 3 initially symptomatic children deceased despite early treatment: one child with OTC-deficiency (aged 9 days) and one child with severe MAD-deficiency (aged 9 months). Within 21 months the study “Newborn Screening 2020” identified additional 28 children with potentially treatable conditions while only marginally increasing the recall rate. Most of these children (89%) were diagnosed presymptomatically. Maternal vitamin B_12_-deficiency was the most frequent finding with 1 in 6000 children affected. Even more children could benefit from screening for the additional target disorders in case of a future comprehensive extension of the German newborn screening panel.

### L-02. Experiences from Israeli Nation-Wide Screening for Primary Immunodeficiencies

SomechRazMD, PhD^1^,^2^1Pediatric Department, Immunology Services; Jeffrey Modell Foundation Center; The Edmond & Lily Safra Children’s Hospital, Sheba Medical Center Tel Hashomer, 52621, Israel2Israeli Society of primary immunodeficiency; The National Lab for Diagnosing SCID—the Israeli newborn screening program

Newborn screening (NBS) programs for Severe Combined Immunodeficiency (SCID), the gravest of primary immunodeficiencies, are being implemented in more and more countries with every passing year. These programs have enabled early diagnosis and prompt treatment of affected infants, and eventually changed the natural history of this devastating disease. The assay that is widely used for detecting affected infants is TREC-based. The latter is a small circle of non-replicating DNA formed as a byproduct during the normal process of T cell receptor (TCR) development. Its level reflects thymic T-cell receptor gene recombination and number of recent thymic emigrants; therefore it is a highly sensitive and specific tool to estimate abnormal T cell immunity such is seeing in patients with SCID. As of October 2015, SCID screening via TREC quantification in Dried Blood Spots (DBS) is part of the Israeli NBS program. The Israeli algorithm uses a commercial kit and consists of a two Guthrie card validation system prior to referral to an immunologist for a full battery of confirmatory tests. The relatively high rate of consanguineous marriages in Israel, especially among non-Jews, correlates with an increased incidence of SCID. Implementation of this successful, lifesaving program allows early diagnosis, prompt treatment and improved outcome of patients, and identify the true incidence of SCID, the causes of many other cases of T-cell lymphopenia and the incidences of SCID and other types of T-cell lymphopenia among different ethnicities.

### L-03. Screening for Primary Immunodeficiencies in Andalucia

De FelipeB.^1^OlbrichP.^1^DelgadoC.^2^LucenaJ. M.^3^SalamancaC.^4^MarquezJ.^5^AguilarE.^6^BernalM.^6^GomezA.I.^6^NethO.^1^1Seccion de Infectología e Inmunodeficiencias, Unidad de Pediatria, Hospital Virgen del Rocío, Sevilla/Instituto de Biomedicina de Sevilla (IBiS), 41013 Sevilla, Spain2Unidad de Metabolopatías, Hospital Universitario Virgen del Rocío, 41013 Sevilla, Spain3Unidad de Inmunología, Hospital Universitario Virgen del Rocío, 41013 Sevilla, Spain4Unidad de Neonatología, Hospital Universitario Virgen de Macarena, 41013 Sevilla, Spain5Unidad de Pediatría, Hospital Virgen de Valme, 41013 Sevilla, Spain6Unidad de Neonatología, Hospital Universitario Virgen del Rocío, 41013 Sevilla, Spain

Andalusia is leading in number of births in Spain with 80,000 per year. In 2014, we initiated the study prospective and observational and longitudinal pilot study of neonatal screening for inmunodeficiencies using a RT–PCR-based T-cell receptor excision circles (TREC)/kappa-recombining excision circles (KREC)/b-actin determination assay. Since then, we have tested the original technique and two commercial kits. Here we provide an overview of our experience with these three methods in our setting. TRECs (all methods) and KRECs (only in the original technique and Roche-TIB™) were determined using dried blood spots from neonates in three public hospitals in Seville, Spain. Internal and external controls (provided by the CDC) were included. From 2012 to 2017, a total of 12,212 DBS samples were analyzed with either one of the three techniques: original (*n* = 8582), Perkin-Elmer™ (*n* = 1026), and Roche-TIB™ (*n* = 2604). All three tested kits correctly identified the samples derived from patients with T and/or B cell deficiencies. Sensitivity and specificity were similar and all methods allowed discernment between healthy subjects, internal controls, and external controls. The original and Roche-TIB method allow the quantifying of TRECS and KRECS, whereas the Perkin-Elmer kit only provides TREC values. T- (and B-) cell lymphopenias were correctly identified by three (two) methods tested.

## 3. Oral Presentations

### O-01. Parents’ Opinions on the Communication of Positive Newborn Screening Results for Cystic Fibrosis in Bavaria

BrockowInkenNennstielUtaBavarian Health and Food Safety Authority (LGL), 85764 Munich-Oberschleißheim, Germany

In Germany, newborn screening for Cystic Fibrosis (CF) was established in September 2016. The risk of psychological harm in families of newborns with false-positive screening results is a concern. We investigated the parents’ opinion about the screening information and their experience in the communication of positive screening results.

In February 2018, we started a retrospective questionnaire survey of all parents with positively screened children in Bavaria. The questionnaire was adapted from a well-established Suisse questionnaire and has been sent to the families after the final diagnostic testing.

Until now, the questionnaire was sent to 150 families, and the response-rate was 65 (43.3%). Of these, 66.2% received general information about the newborn screening mainly by nurses only after birth, and 6.2% couldn’t remember any information. 43.1% were informed about the positive test result by the maternity hospital, 16.9% by a CF-center, and 38.5% by a pediatrician. 61% of the parents complained that the provided information from the maternity hospital was not satisfactory, and 83.3% thought more than 3 days between calling and diagnostic testing was too long.

Short delays until final diagnostic testing and satisfactory information are important. Therefore, parents should be informed directly by a CF-center about positive screening results.

### O-02. Psychological Follow-Up of Patients with Inborn Errors of Metabolism Diagnosed in Newborn Screening at the Department of Pediatrics and Adolescent Medicine, Medical University of Vienna

HerleMarionMöslingerDorotheaZeydaMaximilianGreber-PlatzerSusanneKonstantopoulouVassilikiDepartment of Pediatrics and Adolescent Medicine, Medical University of Vienna, A-1090 Vienna, Austria

Children diagnosed with an inborn error of metabolism via newborn screening and treated at the Department of Pediatrics and Adolescent Medicine at Medical University of Vienna undergo regular psychological examinations. Those examinations focus on psychomotor development or on neurocognitive functioning in school-age children. Furthermore, health-related quality of life and psychosocial development are investigated via questionnaires answered by both parents and children. Depending on disease-related risks, neuropsychological functions (e.g., concentration) are also examined. The psychological monitoring includes the following aspects: orientation on international recommendations, comparison of results over long time periods and between patients with different diagnoses, as well as significance of results regardless of cultural and language differences of the patients. Psychological assessments are routinely performed when the children are 18 months, 3, 6, 10, and 14 years old. These assessments aim to optimize the outcome of individual patients as developmental or other difficulties can be identified and treated early. Moreover, data will be provided for scientific evaluation of both psychological and medical outcomes of the patients, which aim to identify conditions where early diagnosis and treatment including psychological monitoring leads to an optimal benefit for the patients and subsequently the health care system.

### O-03. Newborn Screening and Vitamin Disorders

ChrastinaP.BártlJ.BártováP.HodíkJ.JežováR.PaulováM.PinkasováR.PetránkováV.VláškováH.KošťálováE.HrubáE.JahnováH.JešinaP.KožichV.PeškováK.HonzíkT.Department of Pediatrics and Adolescent Medicine, General University Hospital in Prague and 1st Faculty of Medicine, Charles University, 128 08 Prague, Czech Republic

In June 2016, the Czech national newborn screening program was expanded to include 15 inherited metabolic disorders (IMD). The program allows the direct detection of biotinidase deficiency, and indirectly detection of some treatable B-vitamin deficiencies due to dietary restriction, malabsorption, or an IMD in the child or the mother. Screening for MTHFR deficiency may detect patients with folate and vitamin B12 deficiency, while screening for medium-chain acyl-CoA dehydrogenase (MCAD) deficiency may detect riboflavin and coenzyme Q deficiencies.

Amino acids and acylcarnitines were analyzed by tandem mass spectrometry. Biotinidase activity was measured by a fluorometric assay.

Between July 2016 and December 2017, we analyzed samples from 128,531 newborns. We detected 48 patients with IMDs. Disturbed metabolism of vitamins was detected in 9 patients with partial biotinidase deficiency and in 3 patients with maternal vitamin B12 deficiency. We did not detect any newborn with riboflavin or coenzyme Q deficiency. The treatment with biotin or vitamin B12 prevented development of clinical symptoms in all patients.

Addition of biotinidase activity and of low methionine with second-tier total homocysteine measurement led to increase of the detection rate from 1:3,600 to 1:2,700 and enabled an early and an efficient treatment of affected patients.

**Funding:** Supported by MH CZ-DRO VFN64165.

### O-04. Combined Tandem Mass Spectrometry (TMS) Screening Method for Biotinidase Deficiency, and Sickle Cell Disease (SCD)

KleinJeannette^1^BroseAnnemarie^1^FrömmelClaudia^2^LobitzStephan^3^BlankensteinOliver^1^TurnerCharles^4^DaltonR. Neil^4^1Newborn Screening Laboratory, Charité Universitätsmedizin, 13353 Berlin, Germany2Institute of Laboratory Medicine, Charité Universitätsmedizin, 13353 Berlin, Germany3Department of Pediatric Oncology/Hematology, Amsterdam Street Children’s Hospital, 50735 Cologne, Germany4SpOtOn Clinical Diagnostics, Evelina London Children’s Hospital, London SE1 7EH, UK

Modern newborn screening uses a variety of analytical techniques, e.g., qualitative colorimetry, immunofluorescence, and TMS. With the increasing number of conditions screened, there is growing demand to save valuable patient material and optimize laboratory processes. We report the use of TMS to detect SCD and Biotinidase deficiency simultaneously.

SpotOn Clinical Diagnostics developed a dried blood spot (DBS) TMS method for SCD screening, based on extraction and tryptic digestion of hemoglobin from the DBS and detection of disease-specific peptides [1]. The protocol has been adapted to simultaneously measure biotinidase. Initial incubation with biocytin is followed by tryptic digestion. TMS analysis of Hb peptides and the biotin/biocytin pair identifies SCD status and biotinidase activity.

Samples with a ratio of biotin/biocytin <30% of the daily mean were regarded as suspicious for Biotinidase deficiency. Results correlate very well with the output from our standard colorimetric assay. Within a cohort of 30.000 samples, 8 newborns with SCD and 1 with Biotinidase deficiency were identified, demonstrating the potential of combining simultaneous SCD screening with enzyme testing on a single DBS (3 mm) by TMS.

**Reference**

1. Daniel, Y.A.; Turner, C.; Haynes, R.M.; Hunt, B.J.; Dalton, R.N. Rapid and specific detection of clinically significant haemoglobinopathies using electrospray mass spectrometry-mass spectrometry. BJH **2005**, 130, 635–643.

**Funding:** Financial support by the foundation “Kindness for Kids” is gratefully acknowledged.

### O-05 High-Throughput Identification of Hemoglobinopathies & Thalassemias by HRAM/MS

LukacsZoltan^1^MechtlerThomas^2^SchwarzMarkus^2^KasperDavid C.^2^WiesingerThomas^2^1Newborn Screening and Metabolic Diagnostics Unit, Hamburg University Medical Center, Hamburg, Germany2ARCHIMED Life Science GmbH, Leberstraße 20, 1110 Vienna, Austria

The early diagnosis of haemoglobin disorders becomes more and more important due to higher numbers of affected individuals in the European Union, especially in urban areas (e.g., recent studies showed an incidence of ca. 1 in 2500 newborns in Hamburg and Berlin). In general, genetic disorders related to hemoglobin (Hb) can be classified into two main groups (i) hemoglobinopathies and (ii) thalassemias. Early identification of Hb S (SCD) in homozygous form is critical for preventive therapy. In addition, Hb variants (e.g., Hb -C, -D, and -E) with co-inherited Hb S in a heterozygous form are also of importance in regard to early detection and therapy. Due to single mutations in the beta chain, these variants may differ only by 1 Dalton, resulting in issues for the resolution when traditional tandem mass spectrometry is applied, especially for ion species that demonstrate high m/z ratios (>15+). To tackle this challenge, a novel approach using high resolution mass spectrometry was evaluated. For this purpose, intact Hb chains and the corresponding tryptic digest were mixed in a defined ratio and the mixture was directly analyzed by mass spectrometry. Suitability and accuracy of the novel method were confirmed by preliminary experiments.

### O-06. Simultaneous Detection of Amino Acids, Acylcarnitines and Succinylacetone by Tandem Mass Spectrometry without Butylation: Influence of Succinylacetone Extraction on an Established Non-Derivatized Newborn Screening Procedure

RönickeS.BoruckiK.IsermannB.Newborn Screening, Institute for Clinical Chemistry and Pathobiochemistry, Otto-von-Guericke University Magdeburg, 39120 Magdeburg, Germany

Newborn screening (NBS) for hepatorenal tyrosinemia (tyrosinemia type I [TYR 1]; OMIM 276700) has started in Germany in March 2018. According to the guidelines for TYR 1 screening, newborn blood spot samples are analyzed for succinylacetone (SUAC) as the primary metabolic marker. Several publications describe different methods for the analysis of SUAC from dried blood spots. Unfortunately, current protocols including butylation steps are time-consuming and include the risk for interferences with other analyses. Therefore, we evaluated a time-saving method for SUAC extraction with respect to possible disruption of our established non-derivatized NBS procedure. Dried blood spot specimens from 800 newborns, as well as one positive patient and quality control samples from CDC, were analyzed using a LC-MS/MS assay from ChromSystems (MassChrom: Amino acids/Acylcarnitines, non-derivatized) in combination with novel upgrade assay SUAC. Measurements were performed on API4500 mass spectrometer (SCIEX). Dried blood spots underwent two extraction steps. First, amino acids/acylcarnitines were extracted from the filter paper by agitation at 600 rpm for 20 min with 100 μL of extraction buffer (labeled internal standards, methanol). The supernatant was transferred into a new 96-well microtiter plate. Afterwards, SUAC was extracted from the same punched disc by agitation at 600 rpm for 45 min (45 °C) with 150 μL of extraction buffer (labeled SUAC internal standard, acetonitrile, hydrazine derivate). Using this method, positive controls could be clearly distinguished from normal newborns. Based on results from 800 newborns, a TYR I cut-off value of 1.6 μmol/L SUAC was determined (99.9 Perz.). Comparing single extraction of amino acids/acylcarnitines with double extraction of amino acids/acylcarnitines together with SUAC, we found no differences with respect to measured concentrations. Thus, we did not observe an interference of established screening procedures due to additional SUAC extraction.

### O-07. First PID Screening Experience with a qPCR-Based Trec/Krec-Assay

FingerhutRalphUniversity Children’s Hospital, Swiss Newborn Screening Laboratory and Children’s Research Center, CH-8032 Zurich, Switzerland

Background: Newborn screening (NBS) for SCID has been approved by the Swiss Ministry of Health (BAG) in 2018. During the application, it was decided that SCID screening in Switzerland should not only use T-cell receptor excision circles (TRECs) as the primary marker, but also kappa recombining excision circles (KRECs). However, before the official start in January 2019, we had to determine our cut-off values, and establish the determination of TRECS and KRECS with the approved test system in the NBS laboratory in Zurich.

Results: The test system could easily set up in the routine NBS laboratory, since second-tier DNA testing for Cystic Fibrosis is already part of the Swiss NBS programme since 2011. Necessary additional instrumentation were 2 Quantstudio qPCR systems, Thermocyclers, and a centrifuge with swingout buckets for microtiter plates for the post-PCR area. We calculated our initial cut-offs for TREC and KREC copy numbers from 1500 normal newborn screening samples from term babies. Additionally, we checked the TREC and KREC copy numbers from 100 preterm babies taken at the 4th day of life and the 14th day of life. To proof sensitivity, 10 NBS samples from newborns with confirmed SCID were tested.

### O-08. RareScreen—A Cross-Border Newborn Screening Project Detecting SCID, Hemoglobinopathies, and Familial Hypercholesterolemia

WinterTh.^1^GiżewskaM.^2^,^3^OltarzewskiM.^4^BlankensteinO.^5^PacM.^6^MüllerC.^1^NauckM.^1^1University Medicine Greifswald, Newborn Screening Laboratory, 17475 Greifswald, Germany2Pomorski Uniwersytet Medyczny w Szczecinie, 70-204 Szczecin, Poland3Samodzielny Publiczny Szpital Kliniczny Nr 1 PUM, 70-204 Szczecin, Poland4Instytut Matki i Dziecka, 01-211 Warszawa, Poland5Charité-Universitätsmedizin Berlin, 13353 Berlin, Germany6Instytut “Pomnik-Centrum Zdrowia Dziecka”, 01-211 Warszawa, Poland

Expanding the newborn screening panel and improving medical care is of great interest in our region and was realized in the past by implementing a state-wide screening for Cystic Fibrosis. In the framework of a recently granted Interreg 5a EU project, the cross-border implementation of additional screening disorders is planned in the north-east of Germany (Mecklenburg-Vorpommern, Brandenburg) and in the north-west of Poland (Western Pomerania). Screening of SICD, Hemoglobinopathies and Familial Hypercholesterolemia (FH) will be implemented.

Three screening laboratories (Greifswald, Berlin, Szczecin) are involved, each implementing one of the three disorders and providing results for the other project partners. In Szczecin, SCID screening will be performed, quantifying TREC and KREC by qRT-PCR. The screening for Hemoglobinopathies will be performed at the Charité (Berlin) using Tandem mass spectrometry. Tandem mass spectrometry will also be used for the screening of FH, which will be implemented in Greifswald.

The realization will start in May 2018 and will uphold until the middle of 2020. A total of 100.000 newborns will benefit from the improved health prevention offer. All data and experience gained during this project will be taken into account for discussion concerning the perpetuation of these additional screening disorders at the nationwide level.

### O-09. Early Check: Generating Evidence to Inform Newborn Screening for Rare Disorders

BaileyDonCenter for Newborn Screening, Ethics, and Disability Studies, RTI International, Research Triangle Park, NC 27709 USA

Newborn screening (NBS) is designed for pre-symptomatic identification of conditions for which there are effective treatments that must begin early. Because most conditions considered for NBS are rare, researchers have difficulty identifying enough babies to test the benefits of early identification. We address this gap through Early Check, a voluntary program in which screening for a carefully selected panel of conditions is offered under a research protocol to parents of the 120,000 babies born in North Carolina, USA each year. Our goal is to establish and prove the benefits of an infrastructure to enable research on “not-yet-screened” conditions to better understand (a) effective strategies for “virtual” recruitment and e-consent; (b) parents’ interest in screening; (c) the prevalence and ethnic distribution of targeted conditions; (d) early natural history; and (e) the benefits of early identification. We will launch Early Check in June 2018, initially offering screening for fragile X syndrome and spinal muscular atrophy. This presentation describes our multi-disciplinary approach, project rationale, criteria for selecting conditions, processes for inviting families to participate and consent, plans for short- and long-term follow-up, a telegenetic counseling component, and a new instrument to assess family outcomes of NBS, a key feature of our evaluation plan.

**Funding:** National Center for Advancing Translational Sciences, Eunice Kennedy Shriver National Institute of Child Health and Human Development, The John Merck Fund, Asuragen.

## 4. Poster Presentations

### P-01. Screening for Galactosemia: A Study from Hungarian NBS System

SzatmáriIldikóZsideghPetraBaloghLídiaCenter of Newborn Screening and Inherited Metabolic Disorders, 1st Department of Pediatrics, Semmelweis University, 1083 Budapest, Hungary

Newborn screening (NBS) for galactosemia is done primarily to detect clinically devastating classical galactosemia due to defective function of galactose-1-phosphate uridyltransferase (GALT); however, several other aetiologies may cause elevated total galactose level in the neonatal samples.

Here we report the results of galactosemia screening in the Center of Newborn Screening and Inherited Metabolic Disorders Budapest between 2011 and 2017. A two-tier system is used, with total galactose concentration (PerkinElmer, Total Galactose kit) and enzyme activity of GALT (in-house fluorimetry assay) in dried blood spots (DBS) as primary and secondary markers. Previously defined positive and critical cutoffs for total galactose are 10 mg/dl and 15 mg/dl respectively.

Using the above mentioned fluorimetric method, 420187 DBS samples were analyzed for total galactose level, and 1804 GALT assays were performed. 436 positive samples were measured, out of which 190 were above critical level. 48 cases proved to have low GALT activity and were genetically confirmed, and in 25 cases, portal vein imaging showed the presence of a hepatic shunt.

The primary goal of NBS for galactosemia is to identify GALT-deficient galactosemia. Semiquantitative GALT analysis with an appropriate cutoff identifies these cases; however, analysis of the accumulated data of NBS and clinical expertise may improve follow-up guidelines for non-classic galactosemia as well.

### P-02. Severe Anemia in a Newly Diagnosed Infant with LCHAD Deficiency

ŠmonAndraž^1^TanšekMojca Žerjav^1^BratinaNataša^1^JazbecJanez^1^,^2^LampretBarbka Repič^1^RemecŽiga Iztok^1^BattelinoTadej^1^,^2^GrošeljUrh^1^1University of Children’s Hospital, UMC Ljubljana, 1000 Ljubljana, Slovenia2Faculty of Medicine, University of Ljubljana, 1000 Ljubljana, Slovenia

Mitochondrial trifunctional protein is an enzyme that catalyzes three steps in the mitochondrial fatty acid β-oxidation; one of them is the activity of long-chain 3-hydroxyl acyl-CoA dehydrogenase deficiency (LCHAD). Children with LCHAD deficiency can present with acute hypoglycaemia, liver disorder, lactic acidosis, and cardiomyopathy. On the evening before hospitalization, the child was still normal. The next morning, the child did not wake up for breastfeeding as usual and was less responsive. Upon admission at the hospital, her blood sugar was 1.2 mmol/L and immediately corrected. Ultrasound of head and abdomen were normal. The child had elevated triglycerides, decreased fibrinogen, mildly elevated bilirubin, transaminases, slightly prolonged international normalized ratio (INR) and non-measurable prothrombin. In addition, severe anaemia was detected. LCHAD was confirmed by marked increases in disease-specific acylcarnitines and reduced enzyme activity. To explain severe anaemia also, bone marrow puncture was performed. There were no signs of bone marrow’s applause; the foam macrophages were mildly elevated. She fully clinically recovered. Long-chain fatty acid metabolism was confirmed in a 4 months infant with acute hypoglycaemia, hepatopathy, and encephalopathy. For the first time in LCHADD, severe anaemia, possibly due to bone marrow toxic suppression was observed.

### P-03. Newborn Screening in Slovakia—Overview

KnapkovaMaria^1^DluholuckySvetozar^1^,^2^MachkovaMartina^1^MydlovaZuzana^1^1Newborn Screening Centre Slovak Republic, Children’s University Hospital, 974 09 Banská Bystrica, Slovakia2Faculty of Health, Slovak Medical University, 831 01 Banská Bystrica, Slovakia

Newborn screening (NS) in Slovakia is a successful state preventive program with a 30-year history. The program has generally valid rules but also particular. NS in Slovakia is governed by the law of Ministry of Health Slovak Republic. Screening costs are covered by a health insurance for all newborns in Slovakia. Since 1985, there has been a central laboratory in Slovakia—Newborn Screening Centre Slovak Republic—that analyzed dry blood samples taken on the third day of life. In Slovakia, screening is done for CH, CAH, CF and MS/MS technology for PKU, MCADD, VLCADD, LCHADD, IVA, CPT1 def. CPT2, CACT, MSUD in the basic program. In the large spread, recalls are done for disorders with increased Citruline, Metionine, Tyrosine and Glutamine, for PA/MMA and for SCADD and carnitine deficiency. All positive screening results are reported to the diagnostic centres in the West, Middle and East of Slovakia. Since 1985 newborn screening in Slovakia was examined 1,983,695 newborns with 1195 confirmed positive cases. The incidence of all diseases is 1:1660 liveborns. The most common in Slovakia are CH and PKU, and SCAD, CUD, and MCAD in Rome. No CF in Rome’s population of newborns has been confirmed.

### P-04. Newborn Screening and Childbird outside Health Care Facility/Center in Slovakia

RevtakovaAndrea^1^KnapkovaMaria^1^DluholuckySvetozar^1^,^2^MydlovaZuzana^1^MachkovaMartina^1^1Newborn Screening Centre Slovak Republic, Children’s University Hospital, 974 09 Banská Bystrica, Slovakia2Faculty of Health, Slovak Medical University, 831 01 Banská Bystrica, Slovakia

The newborn screening (NS) program in Slovak Republic (SR) is mandatory for all newborns supervised and supported by the Ministry of Health SR (2012). We have about 58,089 newborns per year (2017). Newborn Screening Centre (NSC) manages and maintains newborns screening in Slovakia. Effectivity, organization and structure of the NCS guarantee cover of all babies born in the country. Dry blood spot is the method used. Deliveries outside of the Maternity Hospital and deliveries abroad are some of the new trends. Mothers of these newborns contact NSC with requests for screening. They are sent dry spot sampling materials, including collecting paper, documentation, and manual. It is important to collect a proper amount of blood to Whatman 903 specimen paper to prevent inappropriate sampling of the blood. NSC analyzes all the deliveries, including deliveries at home and abroad. We are closely monitoring this field for raising evidence—from 12 deliveries (2011) to 138 deliveries (2017). It is important to inform the public about these trends and maintain the correct time period for dry blood spot collection in the first 72–96 h of life. Only this can guarantee appropriate early diagnostics and treatment to allow adequate life to every newborn.

### P-05. Marked Reduction of False-Positives in Austrian Newborn Screening for Cystic Fibrosis After Implementation of Pancreatitis-Associated Protein (PAP) as Second-Tier Test

SchanzerAndrea^1^MetzThomas^1^,^2^RennerSabine^1^KonstantopoulouVassiliki^1^,^2^ZeydaMaximilian^1^,^2^1Clinical Division of Pediatric Pulmology, Allergology and Endocrinology, Department of Pediatrics and Adolescent Medicine, Medical University of Vienna, A-1090 Vienna, Austria.2Austrian Newborn Screening, Department of Pediatrics and Adolescent Medicine, Medical University of Vienna, A-1090 Vienna, Austria.

In Austria newborns have been screened for cystic fibrosis (CF) for nearly 20 years by analyzing Immunoreactive trypsinogen (IRT) from dried blood spot cards (DBS). In May 2017, pancreatitis-associated protein (PAP) analysis was introduced into the screening scheme to reduce the false positive rate (0.8% in the past) while keeping sensitivity high (approx. 95%). MucoPAPII (Dynabio) was used after elevated (65–130 ng/mL) IRT for mature newborns. Above 130 ng/mL IRT, newborns were directly screening positive (safety-net). Cut-offs for PAP were ≥3 and ≥1.6 ng/mL for IRT 65–100 and >100–130 ng/mL, respectively. Screening of 61,847 first DBS from May to December 2017 resulted in 575 (0.92%) tests for PAP, of which 111 were positive. 21 of the second cards (7 not received) were screening-positive, adding to the 36 screening-positive via the safety net. 12 of the screening-positive were confirmed CF cases, 7 died, and 6 are still awaiting diagnosis. False-negatives have not been reported yet. In conclusion, PAP 2nd-tier testing reduced total recall numbers to less than 30% compared to the previous screening procedure, while the frequency of detected cases was similar to that of previous years. Assessment of sensitivity, however, depends on false-negative cases that are not known yet.

### P-06. Decreased Concentrations of Isovaleryl-Carnintine in Patients with Maple Syrup Urine Disease (MSUD)

FingerhutRalph^1^KnerrIna^2^MonavariAhmad^2^BorovickovaIngrid^3^RöschingerWulf^4^1University Children’s Hospital, CH-8032 Zurich, Switzerland2NCIMD TSCUH Dublin, Ireland,3Biochem Dep and NBS Lab, TSCUH Dublin Ireland4Laboratory Becker & Colleagues, Munich, Germany

Newborn screening (NBS) for MSUD is a special challenge since patients can metabolically decompensate rapidly without adequate treatment within the first two weeks of life. The sum of the isobaric amino acids leucine, isoleucine, hydroxyproline (Xle), and Val are used as primary markers. For the confirmation of a positive NBS for MSUD, a second-tier UPLC method for the separation of allo-isoleucine is usually used. From 491 samples of MSUD patients under treatment, we additionally measured the concentration of isovaleryl-carnitine (C5) in dried blood spots (DBS). C5 in MSUD patients was 0.02 ± 0.01 μmol/L (mean ± sd); the reference range in healthy newborns is 0.04–0.49 μmol/L (0.1st–99.9th centile). C5 in 9 NBS samples from patients with confirmed MSUD was in the range of 0.01–0.06 μmol/L. From these samples, we additionally calculated the ratios of Xle/Alanin/C5, and Val/Alanin/C5. The ratio Xle/Ala/C5 proved to be the best indicator for MSUD, with the lowest value of the tp’s being 7.3 times higher than the highest value of 168 fn’s. Val/Ala/C5 was the second best indicator, followed by Xle/Ala and Val/Ala, with the lowest tp’s being 4.1, 3.0, and 1.4 times higher than the highest values from fn’s, respectively. As a proof of concept, we are retrospectively evaluating 5 MSUD cases from Ireland and 12 from Bavaria.

### P-07. Vitamin B12 Deficiency Detected in Newborn Screening—An Important Secondary Finding

KonstantopoulouVassiliki^1^,^2^GöschlBernadette^1^MöslingerDorothea^1^Scholl-BürgiSabine^3^KarallDaniela^3^HuemerMartina^4^,^5^WortmannSaskia B.^6^ErtleFlorian^7^ZeydaMaximilian^1^,^2^1Department of Pediatric and Adolescent Medicine, Clin. Division of Pulmology, Allergology, and Endocrinology, Medical University of Vienna, A-1090 Vienna, Austria2Austrian Newborn Screening, Department of Pediatric and Adolescent Medicine, Medical University of Vienna, A-1090 Vienna, Austria3Department of Paediatrics I, Inherited Metabolic Disorders, Medical University of Innsbruck, A-6020 Innsbruck, Austria4Department of Paediatrics, LKH Bregenz, A-6900 Bregenz, Austria5Division of Metabolism, Children’s Research Center, University Children’s Hospital, CH-8032 Zurich, Switzerland6Department of Pediatric and Adolescent Medicine, Paracelsus University, A-5020 Salzburg, Austria7LKH Villach, Division of Pediatric and Adolescent Medicine, A-9500 Villach, Austria

The neonatal vitamin B12 status depends on that of the mother, and a deficiency can result from causes like malabsorption, autoimmune disorders or—increasingly abundant—vegan nutrition. Vitamin B12 deficiency can result in severe and irreversible neurological damage in children. Therefore, an early detection is highly desirable to initiate treatment to avoid developmental disorders. In newborn screening for organic acidurias, propionylcarnitine is determined, which can also be elevated due to vitamin B12 deficiency. A sensitive marker for intracellular vitamin B12 deficiency is methylmalonic acid (MMA) in urine. We retrospectively analyzed newborns with elevated propionylcarnitine from which MMA was analyzed in urine. In 16 of 17 urines analyzed due to suspicious propionylcarnitine in newborn screening, MMA levels were elevated. 9 of these children suffered from a severe (<135 μmol/L) and 2 from a moderate (135–200 μmol/L) vitamin B12 deficiency; only 5 were unaffected. In conclusion, expanded newborn screening can indicate a strong suspicion for vitamin B12 deficiency. Therefore, urine should urgently be analyzed for MMA after a respective request of the newborn screening lab, and if elevated, vitamin B12 deficiency has to be considered.

### P-08. Maternal Vitamin B12 Deficiency Leads to Elevated Methylmalonate and Total Homocysteine with Normal Propionylcarnitine in Dried Blood Spots from Newborn Screening

TsoukaAlexandra^1^FingerhutRalph^2^BraissantOlivier^3^RouxClothilde^3^BallhausenDiana^1^1Centre des maladies moléculaires, CHUV, Lausanne, Switzerland2NBS & Children’s Research Center, University Children’s Hospital, CH-8032 Zurich, Switzerland3Service of Clinical Chemistry, CHUV, Lausanne, Switzerland

Elevation of methylmalonate in plasma and urine is found in different conditions such as organic acidurias, mitochondrial disorders or nutritive cobalamin deficiency. The most common monogenic cause is methylmalonyl-CoA mutase deficiency. A 6-week-old boy was found to have significantly elevated urinary methylmalonate excretion. Organic acid analysis was performed due to the diagnosis of pernicious anemia and vitamin B12 deficiency in his mother. Pregnancy and birth had been uneventful. The newborn was thriving well under breast milk and had a normal neurological examination. At 6 weeks of life, urinary methylmalonic acid was elevated at 1560 mmol/mol creat. (*N* < 53), 3-OH propionic acid at 24 mmol/mol creat. (*N* < 13) and 2-methylcitric acid at 141 mmol/mol creat. (*N* < 22). Propionylcarnitine was found elevated at 5.49 μmol/L (*N* 0.1–1.77) in plasma acylcarnitine profile. Plasma vitamin B12 concentration was found low at 63 pmol/L (N 326–591) and methylmalonate elevated at 44.94 μmol/L (*N* < 0.28). Opening of newborn screening data of this child showed normal amino acids and acylcarnitines with particularly normal concentrations of methionine and propionylcarnitine. There was thus no evidence for methylmalonic aciduria or a disorder of cobalamin metabolism. However, further analyses of dried blood spots from newborn screening revealed elevated methylmalonate at 455 nmol/L (*N* < 150) and total homocysteine at 57 μmol/L (*N* < 6). The boy was treated with intranasal vitamin B12 spray (1 push = 500 μg once a week). After 6 weeks of treatment (age 3 months), all parameters in blood and urine normalized except methylmalonate in plasma that was still slightly elevated (0.30 μmol/L). We report a case of vitamin B12 deficiency due to maternal pernicious anemia that could have been detected by elevation of methylmalonate and total homocysteine levels in dried blood spots from newborn screening.

### P-09. Screening for Vitamin B12 Deficiency in Austrian Newborns by 2nd-Tier Measurement of Homocysteine

RozmaričTomažLeditznigNadjaMetzThomasKonstantopoulouVassilikiZeydaMaximilianAustrian Newborn Screening, Department of Pediatrics and Adolescent Medicine, Medical University of Vienna, 1090 Vienna, Austria

Early diagnostics and treatment are crucial in preventing irreversible neurological damage caused by vitamin B12 deficiency, which can be indicated by elevated propionylcarnitine values in the screening for organic acidurias in newborns. Though vitamin B12 deficiency screening may be justified due to fulfilling of the Wilson and Jungner criteria, it is rarely listed in regular screening panels or regarded, if at all, as a secondary condition or incidental finding. The aim of this study is to test a screening strategy directly targeting vitamin B12 deficiency together with inborn cobalamin disorders such as CblC, D. Based on published data on elevated homocysteine values in newborns with vitamin B12 deficiency, we recently started to determine homocysteine concentrations after increased propionylcarnitine in Austrian newborns’ dried blood spot samples using tandem mass spectrometry. Until now (1 month of the study, 160 samples analyzed for homocysteine), one patient with elevated homocysteine (14.1 μM; propionylcarnitine was 7.9 μM) was found and turned out to suffer from a moderate vitamin B12 deficiency (157 pg/mL). In conclusion, a screening scheme for vitamin B12 deficiency was implemented in Austrian Newborn Screening and is currently being evaluated.

**Funding:** Supported by the Austrian National Bank (Jubiläumsfonds, Project Nr. 17362 to M.Z.).

### P-10. Evaluation of Newborn Screening for Congenital Adrenal Hyperplasia in Austria Since its Implementation in 2001.

FriedrichMelanie^1^ZeydaMaximilian^2^KonstantopoulouVassiliki^2^RiedlStefan^1^,^3^1Department of Pediatric and Adolescent Medicine, Clin. Division of Pulmology, Allergology, and Endocrinology, Medical University of Vienna, A-1090 Vienna, Austria2Austrian Newborn Screening, Department of Pediatric and Adolescent Medicine, Medical University of Vienna, A-1090 Vienna, Austria3St. Anna Children’s Hospital, Medical University of Vienna, A-1090 Vienna, Austria

Congenital adrenal hyperplasia (CAH) caused by 21-hydroxylase deficiency is an adrenal disorder resulting in androgen excess and decreased cortisol biosynthesis. In order to prevent life-threatening salt loss, newborn screening for CAH by measuring 17-OHP for early diagnosis was introduced in 2001. However, additional reasons for elevated 17-OHP, such as stress, illnesses and prematurity, results in a high rate of false-positive cases. Retrospective analysis of centralized newborn screening data of CAH-positive subjects in Austria (2002–2015) was performed. From 2002–2015, 70 out of 1.102.677 children were diagnosed with confirmed CAH. 3970 newborns had elevated 17-OHP values above weight-adjusted cut-off levels. This resulted in a recall rate of 0.36%. The positive predictive value (PPV) regarding confirmed CAH was 3.43% in terms and 0.49% in preterms. The high recall rate was mainly due to the high percentage of false-positive preterm infants. The preponderance of male newborns in cases of false positives is partly explained by a higher rate of male immaturity. This analysis may serve as a basis for improvement of PPV by adaption of cut-off levels of newborns with a birth weight ≤2.500 g. The distribution of values of true-positives, however, is a considerable challenge of this task.

### P-11. Stability of IRT in Dried Blood Samples

FingerhutRalph^1^NennstielUta^2^1Swiss Newborn Screening Laboratory, and Children`s Research Center, University Children’s Hospital, CH-8032 Zurich, Switzerland2Bavarian Health and Food Safety Authority, Screening Center, Oberschleissheim, Germany

Cystic fibrosis (CF) is one of the most common autosomal recessive disorders with a frequency of about 1 in 3500 livebirths in the white population. Switzerland started CF-NBS in January 2011, and Germany in September 2016. Due to an amendment to the act for Genetic Testing in Humans from 2010, the situation for the German NBS programs became very complicated. Due to the new legal regulations, the testing for CF can only be performed after the parents have been informed by a physician about CF-NBS. This does not only affect the second-tier mutation analysis, but also the primary IRT testing. IRT measurement has to be postponed until this information reaches the laboratory. However, there is so far no information available on the short-term stability of IRT in DBS at ambient temperatures, which makes it rather impossible for NBS laboratories to define correct cut-off values for IRT, which is not immediately measured at the day of arrival in the NBS laboratory. We have confirmed the short-term stability of IRT in 42 left-over DBS. In addition, we also tested the variability of IRT measurements in 500 DBS where the initial IRT was >50 ng/mL whole blood.

### P-12. Seasonal Variation of IRT Values and Sex Differences

MetzThomasKonstantopoulouVassilikiZeydaMaximilianAustrian Newborn Screening, Department of Pediatrics and Adolescent Medicine, Medical University of Vienna, A-1090 Vienna, Austria

Immunoreactive trypsinogen (IRT) is the primary marker for cystic fibrosis screening and due to its frequent elevation in healthy newborns, a cause of substantial numbers of false positives. Therefore, cut-off values have to be carefully considered. Here, we evaluated the influence of the season and sex on IRT values and false-positives over three years and false-negatives over 19 years of cystic fibrosis screening in Austria. We observed a striking decrease of IRT values in the warm season (June–August) reflected by a decrease in the false-positive rate via the static cut-off of 65 ng/mL. False-negatives (*n* = 22), however, did not follow this trend. Furthermore, we observed more false-positive females than males (1.07% vs. 0.80%), while false-negatives were evenly distributed (11/11). In conclusion, neither season nor sex appeared to affect sensitivity of cystic fibrosis screening, while these factors may be considered for optimal reduction of false-positives.

